# Effect of electrophysical resources on healing of neurotendinous injury in an experimental model of type I diabetes and kidney disease

**DOI:** 10.1590/acb370402

**Published:** 2022-06-27

**Authors:** Patrícia Henrique Silva, Pâmela Henrique Silva, Gilberto Gonçalves Facco, Adalberto Vieira Corazza, Josivaldo Godoy da Silva, Iandara Schettert Silva

**Affiliations:** 1 Fellow Master’s degree. Universidade Federal do Mato Grosso do Sul – Postgraduate Program in Health and Development – Campo Grande (MS), Brazil.; 2 Fellow Master’s degree. Universidade Federal do Mato Grosso do Sul – Postgraduate Program in Health and Development – Campo Grande (MS), Brazil.; 3 PhD. Universidade Anhanguera – Postgraduate Program in Environments and Regional Development – Campo Grande (MS), Brazil.; 4 PhD. Universidade Federal do Mato Grosso do Sul – Campo Grande (MS), Brazil.; 5 PhD. Universidade Federal do Mato Grosso do Sul – Postgraduate Program in Health and Development – Campo Grande (MS), Brazil.; 6 PhD. Universidade Federal do Mato Grosso do Sul – Postgraduate Program in Health and Development – Campo Grande (MS), Brazil.

**Keywords:** Renal Insufficiency, Chronic, Low-Level Light Therapy, Cryotherapy, Diabetes Mellitus

## Abstract

**Purpose:**

To evaluate and describe the effect of electrophysical resources laser therapy (LLLT), intravascular laser blood irradiation (ILIB), and cryotherapy on the healing process of neurotendinous injury, as well as possible systemic changes, in the experimental model of type 1 diabetes associated with kidney injury.

**Methods:**

The animals were randomized into four groups: G1) healthy control with untreated injury; G2) healthy control with injury and treatment; G3) disease control with untreated lesion; G4) disease with injury and treatment. Furthermore, the treated groups were divided into three, according to the type of treatment. All animals were induced to neurotendinous injury and treated according to the therapeutic protocols. Healing and inflammation were analyzed by semiquantitative histopathological study.

**Results:**

It was observed in sick animals treated with cryotherapy and ILIB reduction of inflammatory exudate, presence of fibroblasts and organization of collagen, when compared to the effects of LLLT. Moreover, there was reduction in glycemic levels in the group treated with ILIB.

**Conclusions:**

Cryotherapy promoted reduction in inflammatory exudate and organization of collagen fibers, in addition to the absence of signs of tissue necrosis, in the groups treated with and without the disease. ILIB therapy showed the same findings associated with significant reduction in glycemic levels in the group of diseased animals. The application of LLLT showed increased inflammatory exudate, low organization of collagen fibers and low sign of tissue degeneration and necrosis. This study in a model of associated diseases (diabetes and kidney disease) whose effects of electrophysical resources studied after neurotendinous injury allows us to verify histopathological variables suggestive of patients with the same comorbidities.

## Introduction

Diabetes mellitus (DM), a socioeconomic disease, understood as a chronic metabolic syndrome, results from defective or insufficient secretory response of the hormone insulin, resulting in hyperglycemia^1^. This can lead to numerous complications, from delayed tissue repair due to biochemical changes, as well as cardiomyopathies, encephalopathies, neuropathies and nephropathies^1^. These complications affect function and quality of life, resulting in disability and reduced productivity^2^.

Microvascular complications occur in all types of diabetes, which in turn corroborate to the emergence of the main complications that affect diabetic patients. Retinopathy stands out, which causes visual problems, increased incidence of falls and blindness; nephropathy, which in turn causes severe chronic kidney disease, leading to renal replacement therapy and even death; and peripheral neuropathy, which causes orthostatic hypotension, dysautonomia, foot ulcers, and limb amputations^2^.

High blood glucose levels promote activation of protein C kinase, which leads to the production of nitric oxide, promoting oxidative stress, increasing the synthesis of free radicals, which result in ischemic injury and delay in the healing process^3^^-^ 5.

Among the complications arising from the pathological condition, diabetic peripheral neuropathy is a disorder commonly rehabilitated by physical therapists, using physical and phototherapeutic resources, effective in controlling painful process, inflammations, edema, tissue regeneration and repair, revascularization, nutrition, and tissue oxygenation^1^^,^ 5 ^,^ 6.

The therapeutic approach through low-cost physical resources, such as laser therapy, intravascular laser irradiation of blood therapy (ILIB), and cryotherapy, has shown effective results in tissue repair and minimization of tissue damage^7^.

To generate biological effects when reaching the tissues, the laser light must be absorbed by the target tissue. LLLT in healthy tissues is able to produce reactive oxygen species (ROS). However, when applied under conditions of oxidative stress, there is reduction in ROS synthesis^8^.

On the other hand, ILIB therapy, in addition to the expected effects of laser therapy, modulates the signaling of reactive enzymes in the respiratory chain, through mitochondrial components, inducing positive effects on the expression of immunoglobulins, interferons and interleukins, being able to promote an increase in the supply of oxygen to the tissues, ceasing tissue hypoxia, normalizing metabolism, and improving the oxidation of energy transport molecules, glucose and pyruvate, due to the greater production of adenosine triphosphate. Recent studies have pointed out its use in several systemic conditions^8^^,^ 9.

There are data in the literature that prove the power to weaken and delay the infiltration of inflammatory cells after the application of cryotherapy, which can be explained by the vasoconstriction induced by cold, promoting reduction in cell permeability and lymphatic vessels and capillaries, which, in turn, reduces leakage of liquid into the interstitium^10^.

It is believed that the electrophysical resources used in the physiotherapeutic rehabilitation of patients only promote local effects, not being able to interfere with the systemic metabolism of individuals affected by DM and chronic kidney disease.

Therefore, the aim of the present study was to evaluate the effect of electrophysical resources (laser therapy, ILIB and cryotherapy) on tissue healing and possible local and systemic metabolic changes, in an experimental model of diabetes-associated kidney disease, mimicking a diabetic nephropathy.

## Methods

The present study is experimental research using animals. It was approved by the Ethics Committee on the Use of Animals (CEUA) of the Universidade Federal do Mato Grosso do Sul (UFMS), Protocol no. 1.065/2019.

The experiment was conducted at the Laboratory of Experimental Models of Disease of the Faculty of Medicine (FAMED) of the UFMS.

Sixty-four adult female mice (*Mus musculus*) of the Swiss strain were used, weighing approximately 20 g, with an average age of 50 days old.

The animals were accommodated in a ventilated rack with individual transparent polycarbonate microisolators, with direct air injection. During the entire experiment, the animals were kept in an acclimatized room with controlled environmental conditions of temperature, 12-hour light/dark cycle and humidity.

After the establishment of the disease models, they were fed a standard balanced commercial diet specific for the species and access to water *ad libitum*.

The experiment lasted 35 days. The animals were adapted to the environment for seven days before starting the animal disease model protocols.

### Experimental design

**Figure 1:**
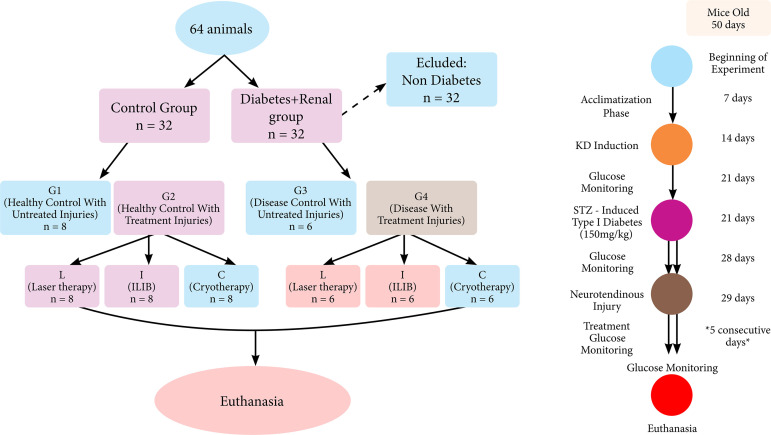
Flowchart of the distribution of animals in groups and protocols. The flowchart shows the random division of animals into groups: G1 (healthy control with untreated injury); G2 (healthy control with injury and treatment), subdivided into subgroups L (low-level laser therapy treatment), I (intravascular laser irradiation of blood treatment), and C (cryotherapy treatment); G3 (disease control with untreated lesion); G4 (disease with injury and treatment), subdivided into subgroups L (low-level laser therapy treatment), I (intravascular laser irradiation of blood treatment), and C (cryotherapy treatment). While the timeline illustrates the phases of the experiment: acclimatization period (seven days), kidney disease induction (14 days), glucose monitoring (21 days), induction of the streptozotocin type I diabetes protocol (21 days), glucose monitoring (28 days), neurotendinous injury protocol (29 days), five days of consecutive treatment, and glucose monitoring and glucose monitoring, before euthanasia (35 days).

ILIB: intravascular laser blood irradiation; KD: kidney disease; STZ: streptozotocin.

The animals were randomized into four groups:

- Group 1 (G1): healthy control with untreated injury (n = 8);
- Group 2 (G2): healthy control with injury and treatment, subdivided into three subgroups, L (low-level laser therapy treatment), n = 8, I (intravascular laser irradiation of blood treatment), n = 8, and C (cryotherapy treatment), n = 8;
- Group 3 (G3): disease control with untreated lesion (n = 6);
- Group 4 (G4): disease with injury and treatment, subdivided into three subgroups, L (low-level laser therapy treatment), n = 6, I (intravascular laser irradiation of blood treatment), n = 6, and C (cryotherapy treatment), n = 6 (Fig. 1).

Groups G3 and G4 were composed of animals induced to the model of kidney disease associated with diabetes. Animals that did not present hyperglycemia (> 200 mg/dL) were excluded from the groups.

### Procedures

#### Diabetes-associated kidney disease

The diabetes-associated kidney disease model was performed according to experiments previously performed at the Laboratory of Experimental Models of Disease of the UFMS, as described ahead:

- Renal ischemia and reperfusion, by aseptic surgery^11^^-^ 13;
- Kidney damage was monitored for 14 days, using urine dipstick tests, in which renal damage was verified by the parameters pH, density, urobilinogen, and urinary glucose, as well as the presence of proteinuria and leukocytes. Renal damage was monitored for 14 days, using a urine reagent strip, by semiquantitative determination of 10 parameters by chemical reaction, in which the kidney was selected by the parameters pH, density, urobilinogen, and urinary glucose, in addition to the presence of proteinuria and leukocytes;
- After 14 days of surgery for induction of kidney disease, the animals comprising the diseased groups were injected intraperitoneally (i.p.) in a single high dose of 150 mg/kg of streptozotocin for induction of diabetes;
- After 2 hours of induction, they began to receive a high-fat Rhoster diet for seven days, with water replaced by aqueous glucose solution (10%) for 24 hours. After this period, they returned to the standard diet of food and water *ad libitum*;
- All animals underwent neurotendinous injury.
- Blood glucose levels were analyzed during the experiment using a digital glucose monitor, and the animals that showed results >200 mg/dL were also considered diabetics;

#### Tibial nerve and common calcaneal tendon injury

**Figure 2:**
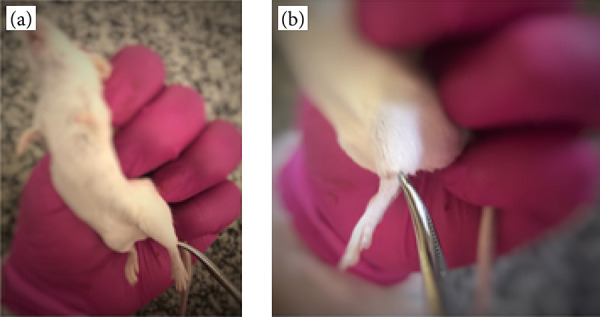
Compression of local left hind limb structures (tibial nerve and common calcaneal tendon). **(a)** Containment. **(b)** Local region compressed by the forceps.

- The neurotendinous injury protocol was performed on the seventh day after the induction and confirmation of the hyperglycemic condition in the animals;
- The animals were submitted to inhalational anesthesia with isoflurane (3-5%), and then manual restraint was performed, holding the skin of the dorsocervical region between the index and thumb fingers and fixing the tail between the little finger of the hand;
- Under inhalation anesthesia with isoflurane (3-5%) and manual restraint, the structures (common calcaneal tendon and tibial nerve) of the left hind limb (LHL) were compressed with a curved Kocher hemostat for 15 seconds (Fig. 2);
- The glycemic levels were checked: before the induction of the DM protocol by streptozotocin; seven days after disease induction and after DM induction and confirmation; after neurotendinous injury and the beginning of the therapeutic protocol; on the fifth and the last day of treatment, to confirm if the animals remained diabetic; the last glycemic measurement was performed before euthanasia to determine whether or not there was a reversal of the diabetic condition.
- At the end of the procedure, they were returned to their individual microisolators. After 24 hours of the traumatic injury, the animals were randomly redistributed between the groups and prepared for the beginning of the therapeutic protocols.

#### Irradiation with low-intensity laser therapy

**Figure 3:**
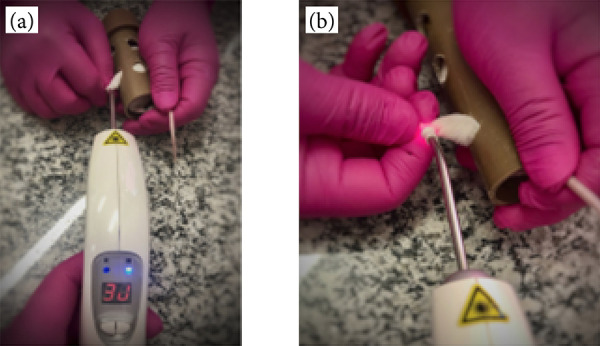
Irradiation with low intensity laser therapy. **(a)** Parameter of energy and application. **(b)** – Direct punctual application. Parameters: 808 nm, continuous, 30 seconds, 100 mW, 3 J/cm^2^, 3 J, 1 cm^2^ area.

The animals in Groups 2 and 4 of subgroup L were submitted to a protocol of low-intensity, infrared laser therapy irradiation, through direct punctual application in the injured area in the LHL, at the frequency of one application daily for five consecutive days (Fig. 3). The therapeutic laser equipment used for the procedure was branded DMC Therapy EC.

#### Intravascular laser irradiation of blood

**Figure 4:**
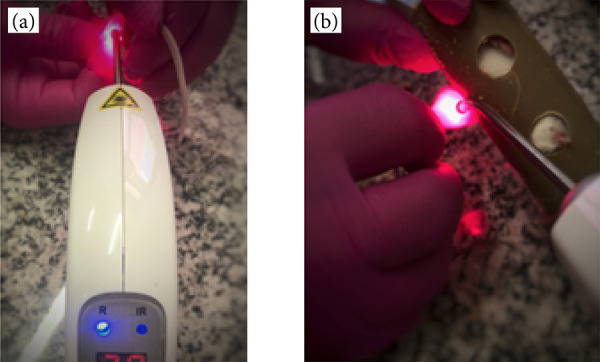
Intravascular blood irradiation with laser. **(a)** Therapeutic laser equipment. **(b)** Direct punctual application in the femoral artery. Parameters: 660 nm, continuous, 360 seconds, 100 mW, 36 J/cm^2^, 36 J, 1 cm^2^ area.

The animals in Groups 2 and 4 of subgroup I were submitted to a protocol of intravascular blood irradiation with red laser, by means of direct punctual application in the region of the femoral artery of the limb contralateral to the lesion, at the frequency of one application daily, for five consecutive days, for a week (Fig. 4). The therapeutic laser equipment used for the procedure was branded DMC Therapy EC.

#### Cryotherapy by immersion

**Figure 5:**
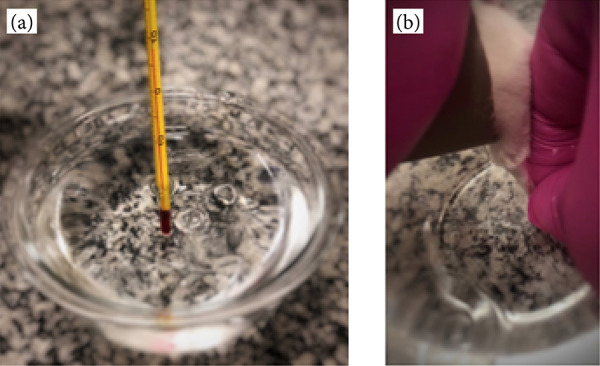
Immersion cryotherapy. **(a)** Average temperature 8-10°C. **(b)** Animal submitted to thecryotherapy protocol.

The animals of Groups 2 and 4 of subgroup C were submitted to a protocol of cryotherapy by immersion, at the frequency of one application daily for five days, in a period of one week. The animal had its injured limb immersed in a container with water and ice, at an average temperature of 8-10°C for 1 minute (Fig. 5).

#### Sample collection and processing

At the end of the experiment, the animals were euthanized with a lethal dose of anesthetic via i.p., ketamine (20 mg/kg)and xylazine (20 mg/kg).

Portions of the tendon and nerve were collected. The piece containing the tendon and nerve was sent for histopathology, fixed in 10% formalin, and later processed for cutting at 5 μm and stained with hematoxylin and eosin (HE).

### Statistical analysis

All data were tabulated and expressed as mean ± standard deviation (SD). After characterizing the variables, based on the histopathological study, a descriptive study of the findings was carried out, in addition to verifying the possible relationships between the variables. Thus, a semiquantitative analysis of the data obtained in the histopathological analysis was performed, based on the images captured from the values were assigned for the analysis according to the frequency of characteristic cells of the inflammatory and healing process. Thus, it was considered:

- Very: 5;
- Frequent: 4;
- Regular: 3;
- Low: 2;
- Absent: 1.

The variables of glycemic levels and semiquantitative analysis of the sections of the anatomopathological pieces were statistically tested by means of one-way analysis of variance, with Tukey’s post-test and Student’s t-test, with significance level determined at p ≤ 0.05, using the program BioEstat 5.3.

## Results

The animal model of kidney disease associated with diabetes in *Mus musculus* mice allowed the study and comparative analysis of the effect of the application of different electrophysical resources in acute conditions in the face of tissue injury.

The glycemic data evaluated over the experimental period allowed us to observe that there were statistically significant differences in the groups at the analyzed moments. There were no statistically significant differences in glycemic levels between the healthy groups (G1, G2C, G2L, and G2 I). Groups G3, G4C, G4L and G4I had statistically significant means higher than groups G1, G2C, G2L, and G2I, at moments M1, M2 and M3, with a significance level of p ≤ 0.05. The G4I (247.0±6.9) showed a statistically significant reduction in glycemic levels at the end of the therapeutic protocol when compared to the other groups G3 (353.3±9.1), G4C (361.3±15.9) and G4L (336.8±15.9). These data are presented in Table 1.

**Table 1:** Weekly glycemic levels. Values expressed as mean ± SD#.

Groups	M0	M1	M2	M3	T Test
G1	117.5±18.0	123.6±16.7a	118.4±13.0a	134.4±20.5a	0.0424^ns^
G2C	113.5±17.2	126.0±21.0	118.3±14.9	130.6±14.5	0.0560^ns^
G2L	108.6±12.1	116.8±15.8	133.1±25.6	127.8±14.1	0.0007*
G2I	111.9±11.0	125.5±13.7	124.5±13.3	135.4±12.5	0.0055*
G3	120.0±8.1	229.7±4.6	296.7±5.3	353.3±9.1	0.0001*
G4C	131.0±20.1	221.8±10.6c	283.5±11.1c	361.3±15.9c	0.0001*
G4L	133.0±19.7	226.8±5.7d	258.5±13.2d	336.8±15.9d	0.0001*
G4I	115.2±11.8	265.8±15.6b	333.2±16.4b	247.0±6.9b	00001*
ANOVA	0.049ns	0.0001*	0.0001*	0.0001*	

SD: standard deviation; ANOVA: analysis of variance;

# results are presented as mean ± SD of mean.

Comparison’s overtime: one-way repeated measures ANOVA, with Tukey’s post-test. The letters expressed in the columns represent the differences in the glycemia of the animals at different times. Comparison between groups: a) statistically significant difference between G1 and G3; b) statistically significant difference between G3 and G4I; c) statistically significant difference between G4C and G4I; d) statistically significant difference between G4L and G4I. Comparisons between diabetic and non-diabetic animals at different times: Student’s t-test. Statistical differences are indicated by

* , p<0.05;

ns values without statistical differences (no significant).

**Figure 6:**
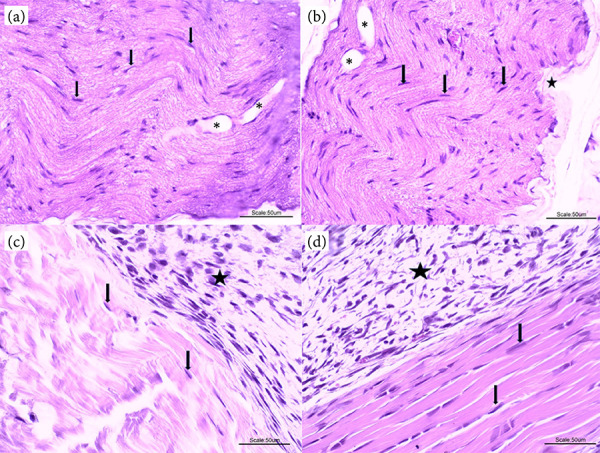
**(a)** (G2C): Photomicrograph of a tendinous segment showing the presence of fibroblasts (arrows) in dense modeled connective tissue. **(b)** (G1): Tendon segment also showing fibroblast nuclei (arrows), dense connective tissue sheath (star) and areas of degeneration (asterisks). **(c)** (G2L) and **(d)** (G2I): Tendon segments showing area of mononuclear inflammatory infiltrate (star) and fibroblast nuclei (arrows). 40x magnification, hematoxylin and eosin staining.

In Fig. 6, it was observed that G2C (Fig. 6a) and G1 (Fig. 6b) presented better morphological analysis, with absence of inflammatory cells, focal areas of degeneration and a certain organization of collagen fibrils. The other groups, G2L (Fig. 6c) and G2I (Fig. 6d), showed mononuclear inflammatory infiltrate (predominantly activated macrophages) in almost all animals, a certain intensity of degeneration and nuclei of active fibroblasts in the peritendinous region. In none of the groups areas of necrosis observed.

**Figure 7:**
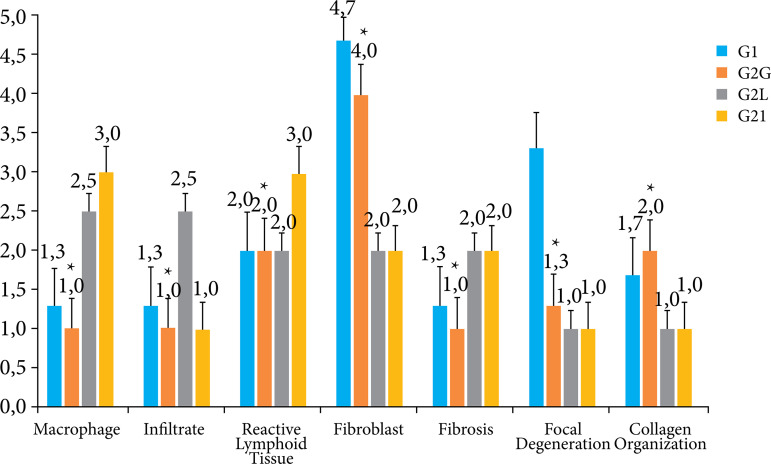
Comparisons between groups: analysis of variance of one-way repeated measures, with Tukey’s post-test. Representation of the semiquantitative analysis of the sections of the anatomopathological parts of the groups (G1, G2C, G2L and G2I). G2C presented better statistically significant results in relation to G1, G2L and G2I, illustrated by the star symbol. Results are presented as mean ± standard deviation of mean.

Intensity of macrophages, infiltrate, reactive lymphoid tissue, fibroblasts, fibrosis, focal degeneration, and collagen organization were observed, with a significant difference between groups G1, G2C, G2L and G2I in all variables when compared with each other, with a significance level of p ≤ 0.05 (Fig. 7).

*Statistical difference, p<0.05.

In the descriptive qualitative histopathological analysis, it was possible to observe the patterns between the groups in relation to the organization of collagen fibers, presence of cellular tissue, inflammatory infiltrate, and signs of degeneration.

**Figure 8:**
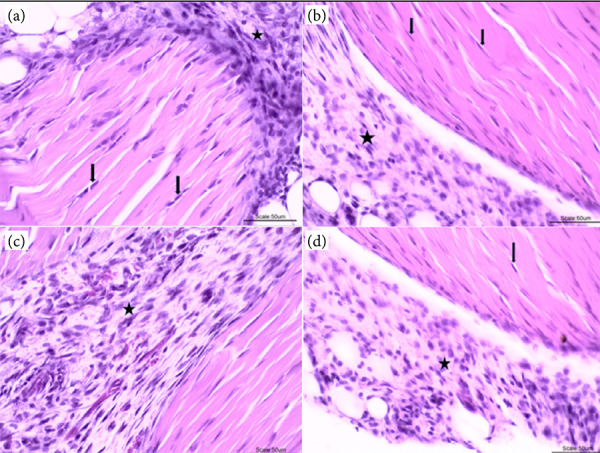
**(a)** (G4C): Photomicrograph of a tendon segment showing the presence of fibroblasts (arrows) in dense patterned connective tissue and an area of peritendinous inflammatory infiltrate (star). **(b)** (G4L): Tendon segment also showing fibroblast nuclei (arrows) and area of peritendinous inflammatory infiltrate (star). **(c)** (G4I) Tendon segment with lower intensity of inflammatory cells (star). **(d)** (G3): Tendon segments showing area of mononuclear inflammatory infiltrate (star) and fibroblast nuclei (arrows). 40x magnification, hematoxylin and eosin coloring.

Figure 8 presents the histopathological results of animals with comorbidities. It was observed that G4I (Fig. 8c) presents a better morphological analysis, with a smaller number of inflammatory cells, present in focal areas, without areas of degeneration and some reorganization of collagen fibrils. The other groups, G4C (Fig. 8a), G4L (Fig. 8b) and G3 (Fig. 8d), showed in almost all animals a mononuclear inflammatory infiltrate (predominantly activated macrophages), in all histological sections, a certain intensity of degeneration, except in G4L, with active fibroblast nuclei, mainly in the peritendinous region. No areas of necrosis were observed in any of the groups.

**Figure 9:**
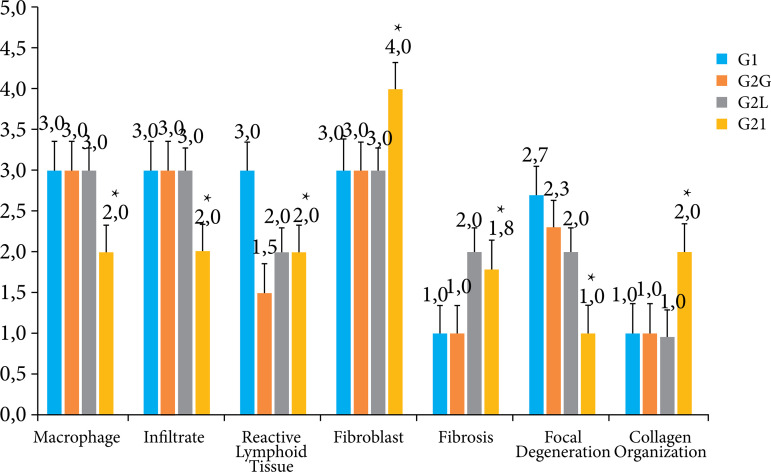
Comparisons between groups: analysis of variance of one-way repeated measures, with Tukey’s post-test. Representation of the semiquantitative analysis of the sections of the anatomopathological parts of the groups (G3, G4C, G4L and G4I). G4I presented better statistically significant results in relation to G3, G4C and G4L, illustrated by the star symbol. Results are presented as mean ± standard deviation of mean.

Significantly different intensity of macrophages, infiltrate, reactive lymphoid tissue, fibroblasts, fibrosis, focal degeneration, and collagen organization were observed between G3, G4C, G4L and G4I, indicating the presence of an inflammatory process and the beginning of the healing process after the injury. The G4I presented better statistically significant results in relation to the variables studied when compared to G3, G4C and G4L, with a significance level of p ≤ 0.05. G4L also presented statistically significant better results when compared to G3 and G4C, when a lower intensity of reactive lymphoid tissue, fibrosis and focal degeneration were observed, with a significance level of p ≤ 0.05. Figure 9 presents a graphical representation of these data.

*Statistical difference, p<0.05.

## Discussion

The literature reports that the induction of the kidney disease model by ischemia and reperfusion, by compression of the renal pedicle, causes local injury to the kidney, that suffers ischemia, and also to the contralateral kidney by the metabolic responses of reperfusion^11^.

Considering that the comorbidity models chosen in this study are, by themselves and separately, highly debilitating for the animals and of low survival, the association of the protocols in this study did not worsen the condition of life and/or survival after the installation of the illnesses.

The feasibility of using mice is due to the feasibility of producing their biological characteristics, in addition to having greater sensitivity to the use of streptozotocin. Previous studies allowed the effective induction of the kidney disease animal model in other animal species of the rodent class^11^^-^ 13.

Patterns between groups were observed and described in relation to the organization of collagen fibers, presence of cellular tissue, inflammatory infiltrate, and signs of degeneration.

DM is a metabolic syndrome characterized, among other signs and symptoms, by hyperglycemia, in other words, high serum glucose. This, in turn, causes microvascular changes that affect various organs and tissues, mainly induced diabetic nephropathy, diabetic neuropathy, and diabetic retinopathy. The healing process of patients with DM is modified by several factors, resulting from the hyperglycemic condition, among them the suppression of inflammatory responses, decrease in angiogenesis and growth factors, alteration in cell proliferation (keratinocytes, fibroblasts and endothelial cells), increased cellular apoptosis and defects in collagen deposition. It is believed that the control of glycemic levels is essential to prevent the emergence of these complications. Therefore, it is necessary that the appropriate treatment is carried out correctly and as soon as possible^14^^,^ 15.

During the normal tissue repair process, fibroblasts are stimulated to proliferate and migrate to the injury site in order to synthesize collagen to restore tissue integrity^14^. In this study, no significant differences were observed in glycemic levels between the healthy groups with lesions (G1, G2C, G2L and G2I), after the application of electrophysical resources, which suggests that the use of resources was not able to promote changes in the healthy organism at a systemic level.

The therapeutic protocol adopted for five consecutive days aimed to analyze the initial effects of the application of different electrotherapeutic resources: immersion cryotherapy, LLLT and ILIB, in the tissue healing process in healthy organisms and patients with kidney disease associated with DM. In the literature, there is still little described about the effects of the application of these resources, in view of the conditions of these associated metabolic disorders^10^^,^ 14.

In this study, animals treated with LLLT showed similar results to animals in the control groups. G2L, of healthy animals with neurotendinous injury, treated with LLLT (808 nm, 3J/cm^2^, 30 s, five consecutive days), at the end of the therapeutic protocol, presented mononuclear inflammatory infiltrate, with predominance of macrophages, small signs of degeneration, active fibroblasts nuclei, mainly in the paratendinous region, with some disorganization of collagen fibrils. These findings corroborate the study by Carvalho et al.^1^, in which the investigators observed, at the end of a period of three days of consecutive treatment with LLLT (660 nm, 10 J/cm^2^), the presence of diffuse mononuclear inflammatory exudate, of granulation with moderate amount of newly formed vessels and disorganized arrangement of fibroblasts and collagen fibers. These results were also similar to G4L ones, a model of kidney disease and associated diabetes, which received LLLT treatment following the same therapeutic parameters and protocols, and the presence of persistent inflammatory infiltrate was also observed, with the presence of macrophages and minimal signs of degeneration, common to the diabetic picture.

In a review study performed by Tomé et al.^8^, treatment in diabetic patients with ILIB therapy was efficient in reducing serum glucose levels in patients with type 2 DM. The protocol adopted was the punctual application of red laser (630 nm) for 30 minutes for 14 consecutive sessions, and the patients had their glycemic levels measured by digital monitoring.

The cryotherapy protocol by immersion (60 s, 8-10°C, five consecutive days) adopted presented statistically significant results in the semiquantitative histopathological analysis when comparing G2C with G1, G2L and GI. The time chosen was sufficient to cool the area according to the literary findings. These corroborate the study by Furtado et al.^10^, who applied in their study the protocol of immersion in cold water for five consecutive days, after a single session of exhaustive exercise, with the temperature of 12°C, for 12 minutes, immersing the whole body. They also demonstrated that treatment with immersion in cold water after a session of intense exercise was able to decrease the formation and damage of ROS and increase cell viability, favoring a faster recovery of muscle tissue. When observing the disease model groups, there was no significant difference in relation to the other treated and untreated groups.

The choice of ILIB therapy in the present study was based on its prevalent use in diabetic patients, as shown in the review by Tomé et al.^8^. It was also observed that ILIB is capable of promoting reduction in the levels of pro-inflammatory interleukin 1 and interleukin 6 and increase in the levels of anti-inflammatory interleukin 10. In this study, we observed that, after the application of ILIB therapy, there was a statistically significant reduction in inflammatory parameters of G4I when compared to the other groups, with significance of p≤0.05. These results were compatible with the histopathological semiquantitative analysis, in which the same group presented better results regarding the tissue healing process, which enhances and contributes positively to the findings in the literature regarding the benefits of this innovative therapy.

In DM, there is low level serum insulin and high levels of fatty acids. These are oxidized by the liver, producing ketone bodies–acetone, acetoacetate and beta-hydroxybutyrate. This process reduces the production of arginine, responsible for the release of insulin, glucagon, adrenal catecholamines, prolactin and growth hormone, deregulating endothelial cells and increasing pro-inflammatory cytokines. Therapy with ILIB has shown significant reduction in arginase and epidermal growth factor receptor, reduction of neuroinflammation and secondary damage according to the literature^8^.

The diversity and lack of alignment of parameters for the use of ILIB therapy make it difficult to standardize and recognize the best modes of application for the different systemic comorbidities. However, despite the heterogeneity between the parameters of use, studies have shown satisfactory results in the clinical condition of patients, with regard to the modulation of inflammation and reduction of levels of pro-inflammatory cytokines, as revealed in the study by Tomé et al.^8^.

Recent studies revealed that the application of ILIB therapy in chronic pathological conditions, such as diabetes and kidney injury, promoted beneficial effects in the clinical condition of these individuals, being associated with increased oxygen availability, reduction of carbon dioxide pressure, tissue removal hypoxia, normalization of tissue metabolism, release of cytokines, modulation of the production of growth factors, and development of new blood vessels^9^.

Razzaghi et al.^9^, in their study with patients with acute kidney injury, administered ILIB to patients (450 nm, 1.5 mW, continuous, 30 min), through a local catheter, for three sessions on alternate days, obtaining reduction in the levels of neutrophil gelatinase-associated lipocalin in urine and plasma, and normal serum creatinine levels, indicating improvement in renal function.

This study suggests a beneficial effect of ILIB on the inflammatory and healing process of neurotendinous injury in a diabetic and renal model. The limitation of this study is the lack of description of the effects of the ILIB, as well as the fact that it is a device that does not allow changes in dosimetry. The other resources used are described in the literature, but, as an option for treatment and rehabilitation in patients with such comorbidities, there is no longer any therapeutic justification^16^.

The strengths of the experimental research were the design of the dual animal model of disease and the non-surgical protocol for neurotendinous injury, as well as the design of therapeutic protocols that allowed us to observe findings indicative of better tissue recovery rates in the phase of acute after application of immersion cryotherapy and ILIB therapy, when compared with application of LLLT.

The non-surgical protocol for neurotendinous injury, in a model of diseases commonly found as comorbidities for patient’s recovery, as well as the design of therapeutic protocols from the daily clinic, allowed us to observe findings indicative of better tissue recovery rates in its acute phase. The lesion phase after application of immersion cryotherapy and ILIB therapy, when compared to the application of LLLT, are consistent with what is often observed in patients.

Both pathologies (DM + kidney disease) promote systemic metabolic changes, deficit in blood supply and reduction in oxygen supply, which negatively interfere in the healing process and tissue repair. Based on this assumption, it is necessary to carry out new studies at different times to verify the effects of resources in the long term.

## Conclusions

Cryotherapy promoted reduction in inflammatory exudate and organization of collagen fibers, in addition to the absence of signs of tissue necrosis, in the groups treated with and without the disease.

ILIB therapy showed reduction in inflammatory exudate and organization of collagen fibers, in addition to the absence of signs of tissue necrosis in the group of sick animals. The animals in this group also showed significant reduction in glycemic levels during treatment.

The application of LLLT showed increased inflammatory exudate, low organization of collagen fibers and low sign of tissue degeneration and necrosis.

This study, in a model of associated diseases (DM and kidney disease) whose effects of electrophysical resources studied after neurotendinous injury, allowed us to verify histopathological variables suggestive of patients with the same comorbidities.
